# SNR enhanced high-speed two-photon microscopy using a pulse picker and time gating detection

**DOI:** 10.1038/s41598-023-41270-7

**Published:** 2023-08-30

**Authors:** Jeonggeun Song, Juehyung Kang, Ungyo Kang, Hyeong Soo Nam, Hyun Jung Kim, Ryeong Hyeon Kim, Jin Won Kim, Hongki Yoo

**Affiliations:** 1 Department of Mechanical Engineering, Korea Advanced Institute of Science and Technology, 291 Daehak-Ro, Daejeon, 34141 South Korea; 2 Department of Biomedical Engineering, Hanyang University, 222 Wangsimni-Ro, Seoul, 04763 Republic of Korea; 3 Cardiovascular Center, Korea University Guro Hospital, 148 Gurodong-Ro, Seoul, 08308 South Korea

**Keywords:** Microscopy, Optics and photonics

## Abstract

Two-photon microscopy (TPM) is an attractive biomedical imaging method due to its large penetration depth and optical sectioning capability. In particular, label-free autofluorescence imaging offers various advantages for imaging biological samples. However, relatively low intensity of autofluorescence leads to low signal-to-noise ratio (SNR), causing practical challenges for imaging biological samples. In this study, we present TPM using a pulse picker to utilize low pulse repetition rate of femtosecond pulsed laser to increase the pulse peak power of the excitation source leading to higher emission of two-photon fluorescence with the same average illumination power. Stronger autofluorescence emission allowed us to obtain higher SNR images of arterial and liver tissues. In addition, by applying the time gating detection method to the pulse signals obtained by TPM, we were able to significantly reduce the background noise of two-photon images. As a result, our TPM system using the pulsed light source with a 19 times lower repetition rate allowed us to obtain the same SNR image more than 19 times faster with the same average power. Although high pulse energy can increase the photobleaching, we also observed that high-speed imaging with low total illumination energy can mitigate the photobleaching effect to a level similar to that of conventional illumination with a high repetition rate. We anticipate that this simple approach will provide guidance for SNR enhancement with high-speed imaging in TPM as well as other nonlinear microscopy.

## Introduction

Two-photon microscopy (TPM) is an attractive biomedical imaging method due to its large penetration depth^1^ and optical sectioning capability^2^. Due to these characteristics of TPM, it is actively used in biological imaging^3–5^. While TPM is widely used with exogenous fluorescent agents for targeting specific biochemical and molecular information^6,7^ or increasing image contrast^8^, it is also used to detect autofluorescence of intrinsic fluorophores from biological samples^9,10^. The label-free imaging is especially advantageous for in vivo imaging and medical diagnostic imaging because it can avoid certain limitations due to the use of exogenous fluorescent agents^8,11^, such as targeting error^12^, toxicity^13^ and complex staining steps^14,15^. Therefore, TPM is widely used in label-free biological imaging to target intrinsic fluorescent molecules such as NADH, FAD^16^, collagen and elastin^17,18^. TPM is known to aid in clinical diagnosis such as diagnosis of atherosclerotic plaque^19,20^, liver fibrosis^21,22^ and cancer^23^. Two-photon microscopy is not limited to clinical diagnosis, but is also used for cell imaging^24^ to observe biological phenomena.

In TPM, nonlinear absorption of two photons occurs to excite fluorophore and emit fluorescence signals. Due to the low probability of the nonlinear interaction of two photons, a pulsed laser with a high peak power is generally used as a light source. Ti: Sapphire femtosecond laser is considered as one of the ideal light sources for TPM for biomedical applications, due to its ultrafast pulse of around 100 fs, a high repetition rate of around 80 MHz, a sufficient average power of around 1 W, and a wavelength of 780–920 nm for UV to blue excitation^25–30^. While Ti: Sapphire femtosecond lasers are successfully used for two-photon imaging, the repetition rate of 80 MHz may not be optimal. In fact, this may be inefficient because the emission intensity of TPM is proportional to the square of the peak power of the excitation pulse. When the pulse repetition rate of the light sources is high, increase in peak power is limited as it will result in increase of the total average power illuminated on the sample, which would cause photodamage^31^ or even ablation^32^ in extreme cases. Even so, an increase in peak power is required for higher intensity of fluorescence emission as autofluorescence in biological samples is relatively weaker than exogenous fluorochromes and produce lower intensity of fluorescence emission. High intensity of fluorescence emission is needed for good two-photon image quality as high intensity signals result in improvement in SNR. In fact, recent studies suggest use of low repetition rate pulsed sources in TPM for signal improvement^33–35^, and reduction in photodamage^36,37^. In addition, M. Clark et al. successfully demonstrated sensitivity improvement and minimized phototoxicity in nonlinear optical microscopy^38^.

A laser scanning microscope produces each pixel of fluorescence image using a single-pixel detector, such as a PMT. Typically, the detector output, i.e., the current or voltage signal, is digitized by a digitizer and the image pixel intensity is determined by integrating a series of data samples during pixel dwell time^39^. In the majority of previous two-photon microscope studies, the fluorescence signal was digitized using a digitizer with an amplifier^3,40,41^. Notably, two-photon fluorescence emission is naturally a pulse that is synchronized with the pulsed illumination. When digitized at high speed, each fluorescence signal occupies only a small fraction of the pixel dwell time. Therefore, the image pixel intensity can be obtained by integrating only the short width of a time gate that contains the fluorescence signal data series without unwanted background noise signals.

In this paper, we report the combined use of the light source with a low pulse repetition rate and time gating detection to enhance the signal of high-speed TPM. A femtosecond Ti: Sapphire laser was used as a light source and a pulse picker was used to control the repetition rate of the pulsed laser source. By reducing the repetition rate of the pulsed laser, higher peak power pulses were achieved to excite the sample with the same average power. As two-photon fluorescence intensity is proportional to the square of the excitation pulse peak power, higher levels of two-photon fluorescence intensity can be obtained with the same average illumination power, resulting in higher SNR two-photon images in a short acquisition time. In addition, the time gating detection method, synchronized with the pulsed illumination, was applied to the acquired pulse data to obtain the fluorescence pulsed signal while excluding background noise. Decreasing the number of time gates reduces the amount of background noise present in the image. Therefore, the combination of a light source with a low repetition rate light source and time gating detection method, which requires fewer time gates, provides more effective suppression of background noise than a high repetition rate light source. One concern about this method is increase of photobleaching, since high pulse energy can increase the photobleaching effect when the nonlinear photobleaching, which is known to be proportional to at least 2nd order of pulse energy, is dominant^42,43^. Although high pulse energy with a low repetition rate can increase the photobleaching, we observed that high-speed imaging with low total illumination energy can mitigate the photobleaching effect to a level similar to that of conventional illumination with a high repetition rate.

## Results

### Signal-to-noise ratio analysis according to repetition rate

The number of photons absorbed by fluorophore per pulse, ${n}_{\alpha }$, in two-photon excitation can be expressed as follows^17,44^:

1 $${n}_{\alpha }\propto \frac{{p}_{0}^{2}}{{\tau }_{p}{f}_{p}^{2}}{\left(\frac{{NA}^{2}}{2hc\lambda }\right)}^{2}$$

where ${p}_{0}$ is the average laser power, ${f}_{p}$ is the repetition rate of the laser, ${\tau }_{p}$ is the pulse width, $\lambda$ is the excitation wavelength, NA is the numerical aperture of the objective lens, $h$ is Planck’s constant and $c$ is the speed of light. If we keep the average power ${p}_{0}$ equal along with other constants in the equation, ${n}_{\alpha }$ according to the pulse repetition rate can be simplified as,

2 $${n}_{\alpha }\propto \frac{1}{{f}_{p}^{2}}$$

As ${n}_{\alpha }$ is proportional to the fluorescence emission per pulse, we can expect the intensity of fluorescence emission, ${I}_{f}$ is the intensity of fluorescence emission resulting from two-photon excitation. ${I}_{f}$, is proportional to the multiplication of the pulse repetition rate, and the number of photons absorbed per pulse:

3 $${I}_{f} {{\propto n}_{\alpha }\times f}_{p}$$

4 $${I}_{f} \propto \frac{1}{{f}_{p}}$$

Therefore, when the average power of the pulsed laser is the same, the intensity of fluorescence emission by two-photon absorption is inversely proportional to the pulse repetition rate.

On the other hand, signal-to-noise ratio (SNR) of image is generally defined as the ratio of the mean signal value to the standard deviation of the background, often expressed as the signal-to-standard-deviation ratio (SSR)^45^. Alternatively, the ratio of mean signal value to the standard deviation of both signal and background, expressed as the signal-to-both-standard-deviation (SSDR)^45^ can be also used to represent SNR of image. SSR and SSDR can be expressed as following equations:

5 $$SSR= \frac{\overline{{I }_{s}}}{{\sigma }_{bg}}$$

6 $$SSDR=\frac{\overline{{I }_{s}}}{{\sigma }_{s}+{\sigma }_{bg}}$$

where $\overline{{I }_{s}}$ and ${\sigma }_{s}$ are the mean and standard deviation of detected fluorescence signal, respectively, and ${\sigma }_{bg}$ is the standard deviation of background. $\overline{{I }_{s}}$ can be obtained by subtracting the background from the fluorescence image. ${\sigma }_{bg}$ in Eq. (5) can be obtained by calculating standard deviation of the background, and ${\sigma }_{s}+{\sigma }_{bg}$ in Eq. (6) can be obtained by calculating standard deviation of the fluorescence image. SSR allows us to estimate the contrast of background and sample images, and SSDR allows us to estimate the degree of separation between fluorescence signal level and noise level^45^. ${\sigma }_{s}$, which is dominated by detector shot noise of signal current, is proportional to the square root of the number of photons detected by photo detector. ${I}_{s}$ and ${\sigma }_{s}$ can be expressed by the intensity of fluorescence emission from fluorophore. SSR and SSDR can be expressed as follows:

7 $${I}_{s}=\alpha \mu {I}_{f}, {\sigma }_{s}= \mu \sqrt{2e{I}_{f}B}$$

8 $$SSR\propto \frac{\alpha \mu {I}_{f}}{{\sigma }_{bg}}$$

9 $$SSDR\propto \frac{\alpha \mu {I}_{f}}{\mu \sqrt{2e{I}_{f}B}+{\sigma }_{bg}}$$

where $\alpha$ is the detector collection efficiency, $\mu$ is the detector gain, $e$ is the electron charge, and B is the bandwidth of the detector. In the case of a high contrast image, we can approximate ${\sigma }_{s}+{\sigma }_{bg}$ to ${\sigma }_{s}$ in Eq. (6) because ${\sigma }_{bg}$, which is detector dark noise, is much smaller than ${\sigma }_{s}$, resulting in

10 $$SSDR\propto \frac{\alpha \mu {I}_{f}}{\mu \sqrt{2e{I}_{f}B}} . \left({\sigma }_{s}\gg {\sigma }_{bg}\right)$$

By simplifying all terms to the intensity of detected fluorescence emission, we can note following relationship.

11 $$SSR\propto {I}_{f}$$

12 $$SSDR\propto \sqrt{{I}_{f}}$$

The SNR of the image is proportional to the square root of the intensity of detected light. From Eqs. (4), (11) and (12), we can calculate the expected SSR and SSDR at different repetition rate by,

13 $$SSR\propto \frac{1}{{f}_{\mathrm{p}}}$$

14 $$SSDR\propto \sqrt{\frac{1}{{f}_{p}}}$$

Therefore, SSR and SSDR is inversely proportional to the pulse repetition rate and square root of the pulse repetition rate, respectively.

**Figure 1:**
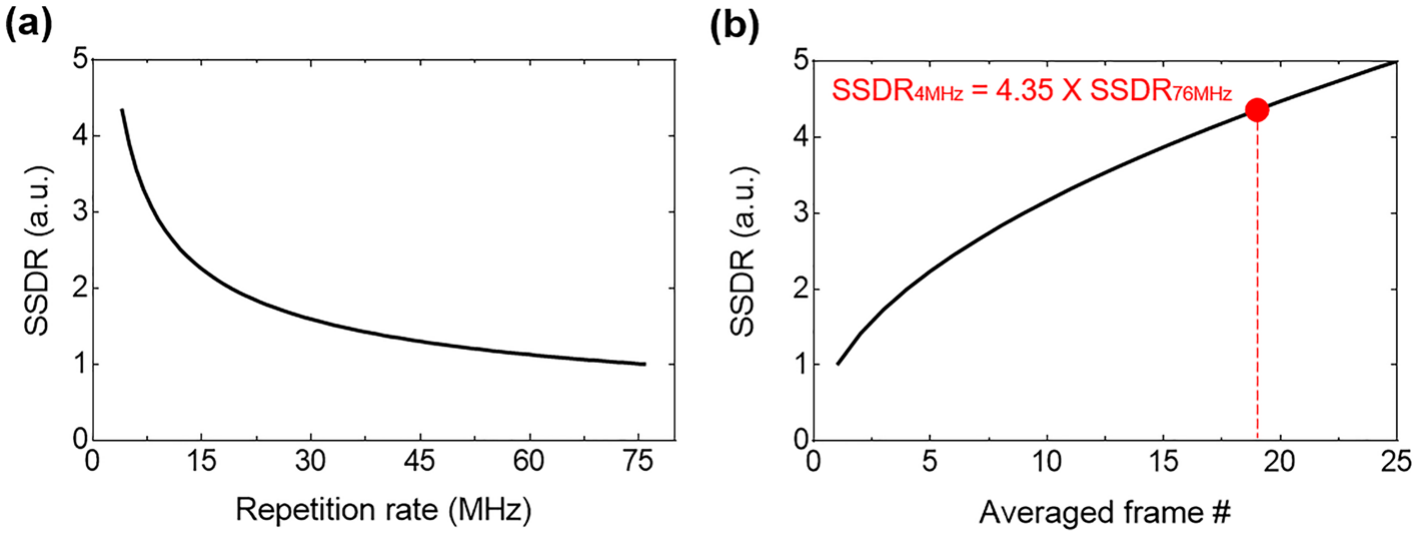
(**a**) Both standard deviation ratio (SSDR) of two-photon excitation fluorescence signal as a function of the pulse repetition rate at the constant average power on the sample. (**b**) The plot of SSDR with a pulse repetition rate of 76 MHz versus the number of frames used in the averaging method. The solid red circle represents the number of frames used in the averaging method for the same SSDR at a pulse repetition rate of 4 MHz.

On the other hand, by averaging multiple image frames, we can enhance the image SNR by reducing both ${\sigma }_{s}$ and ${\sigma }_{bg}$.^46,47^ The improvement of SSDR is proportional to the square root of the number of images used in averaging method. Simply, the SSDR of the averaged image can be expressed as the following equation:

15 $$SSD{R}_{N}=SSD{R}_{1} \times \sqrt{N}$$

where N is the number of images averaged, ${\mathrm{SSDR}}_{\mathrm{N}}$ is SSDR of image averaged N images, and ${\mathrm{SSDR}}_{1}$ is the SSDR of a single image. From Eqs. (14) and (15), we can estimate the required number of frames to acquire equal quality image using low pulse repetition rate source. Figure 1a shows the relative SSDRs of two-photon images at the constant average power on the sample according to the pulse repetition rate. The SSDR values are normalized by the SSDR of conventional two-photon fluorescence images using high pulse repetition rate of 76 MHz. The imaging system using 4 MHz pulse repetition rate is expected to achieve SSDR of 4.3 times higher than that of conventional system. Based on the results above, 19 successive images need to be averaged to obtain the same SSDR as the image using 4 MHz pulse repetition rate at the same average power as shown in the red dot of Fig. 1b. Figure 1 shows that the same SNR can be expected if the same amount of fluorescence emission is utilized to form an averaged image. Therefore, we expect that by reducing the pulse repetition rate from 76 to 4 MHz while keeping the average power on sample, we can get images either with 4.3 times higher SNR or we can get images 19 times faster with the same SNR.

### Two-photon microscope imaging of fluorescent slide

**Figure 2:**
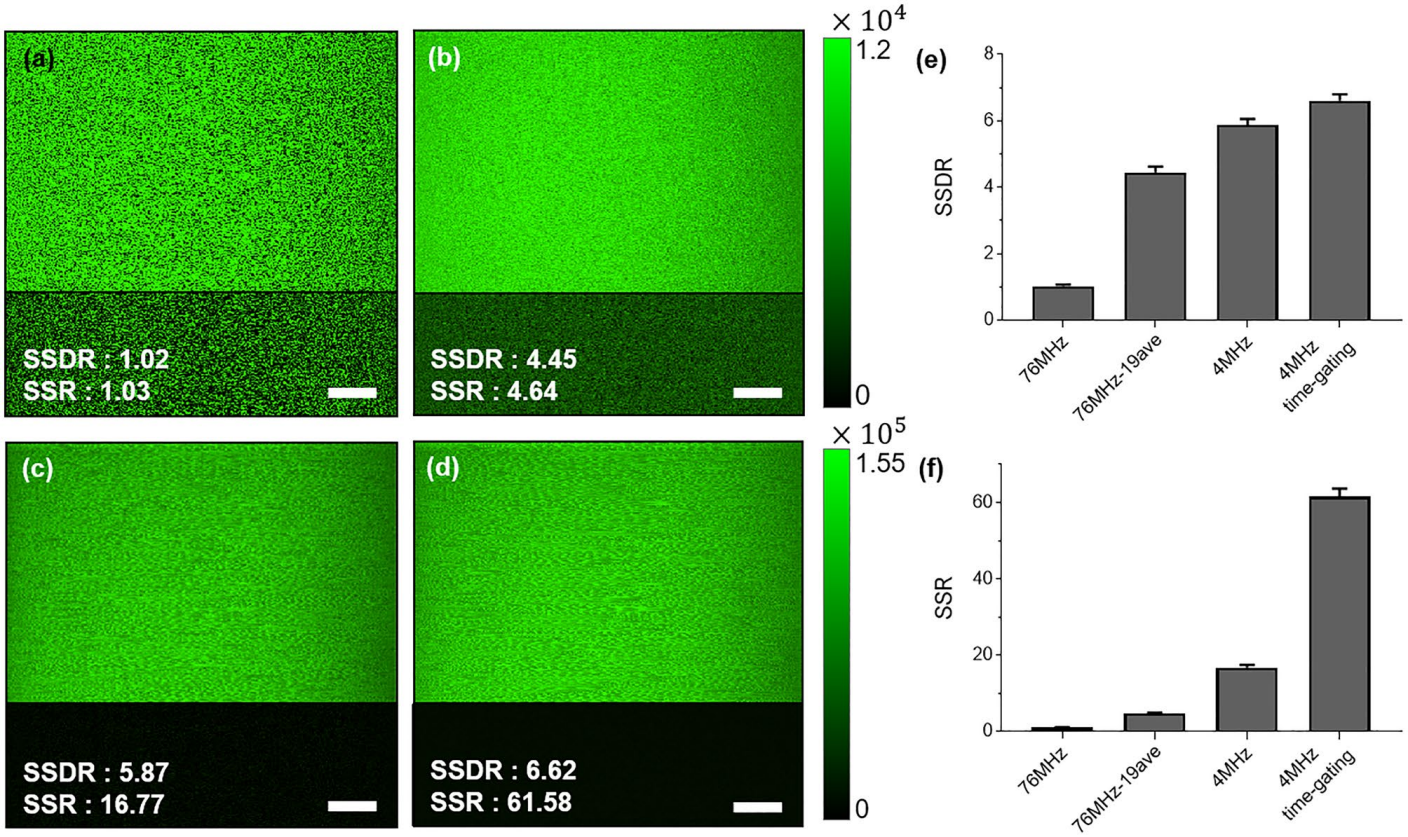
Two-photon fluorescence intensity imaging of the fluorescent slide (FSK2, Thorlabs) with a pulse repetition rate of (**a**) 76 MHz, (**b**) 76 MHz with 19 frames averaged, (**c**) 4 MHz, and (**d**) 4 MHz and time gating method. Note that fluorescence intensity is color-coded in green, and the intensity scale has been adjusted to show a wider range of intensity. The insets (lower part images) show background images of each cases. The scale bars in all images are 50 µm. The bar charts with standard deviation show (**e**) the signal to both standard deviation ratio (SSDR), and (**f**) the signal to standard deviation ratio (SSR) of fluorescence images in (**a**–**d**).

The relationship between the pulse repetition rate and SNR of two-photon fluorescence images was analyzed and validated. Figure 2a–d show the two-photon fluorescence images of the fluorescent slide with the background images located at the bottom of each fluorescence images. The background images were taken without any sample. SSDR and SSR are shown at the bottom of each figures. Figure 2a is the fluorescent slide image using a pulsed source with a high repetition rate of 76 MHz. Figure 2b shows the averaged two-photon fluorescence image utilizing successively acquired 19 fluorescence frames using 76 MHz pulse repetition rate for the similar SSDR to the image using a low pulse repetition rate of 4 MHz. Figure 2c shows the fluorescence image with a pulse repetition rate of 4 MHz. Figure 2d shows the fluorescence image of Fig. 2c after applying the time gating detection method. The time gating windows of 25 ns width allow us to significantly reduce the background dark noise, resulting in high SSR improvement. However, we did not apply the time gating detection method to images captured at a repetition rate of 76 MHz (Fig. 2a and b), because the fluorescence signals are too dense for time gating detection to be employed. The on-sample power was the same as 8.5 mW for all experiments. The frame rate was 7.8 fps with 512 × 512 pixels for both 76 MHz and 4 MHz.

Figure 2e and f show the bar charts for SSDR and SSR according to the various methods in Fig. 2a–d with standard deviation measured by 10 image acquisitions. Mean SSDR of images in Fig. 2a–d are 1.02, 4.45, 5.87, and 6.62, respectively, and mean SSR are 1.03, 4.64, 16.77 and 61.58, respectively. Due to the high fluorescence emission from the fluorescent slide, the SSDR in Fig. 2a–d is higher than 1.0, meaning that signal is well separated from the noise. Due to the higher fluorescence emission caused by low repetition rate of the pulsed source, SSDR in Fig. 2c is 5.75 times higher than Fig. 2a even using the same average power. Averaging 19 frames improves SSDR and SSR in Fig. 2b by 4.36 and 4.50 times, respectively, compared to Fig. 2a. However, the SSDR of Fig. 2c is higher than that of Fig. 2b, even though Fig. 2b and Fig. 2c utilize the same amount of the fluorescence emission to form an image. This is due to the background noise in Eq. (9), which was not negligible. As a result, it would take more than 19 times the acquisition time to achieve the same level of the image quality as Fig. 2c. Since SSDR is dominated by a shot noise, the time gate detection effect is limited in the improvement of SSDR in Fig. 2d, also shown in Fig. 2e. However, time-gating detection method significantly increases SSR since the SSR is dominated by a dark noise. By applying time gating detection method, the average dark noise level was reduced to about one-tenth, improving the SSR by 3.67 times compared to unused. The resultant SSR is about 60 times higher compared to conventional method with high repetition rate.

### Two-photon microscope imaging of a swine vascular imaging

**Figure 3:**
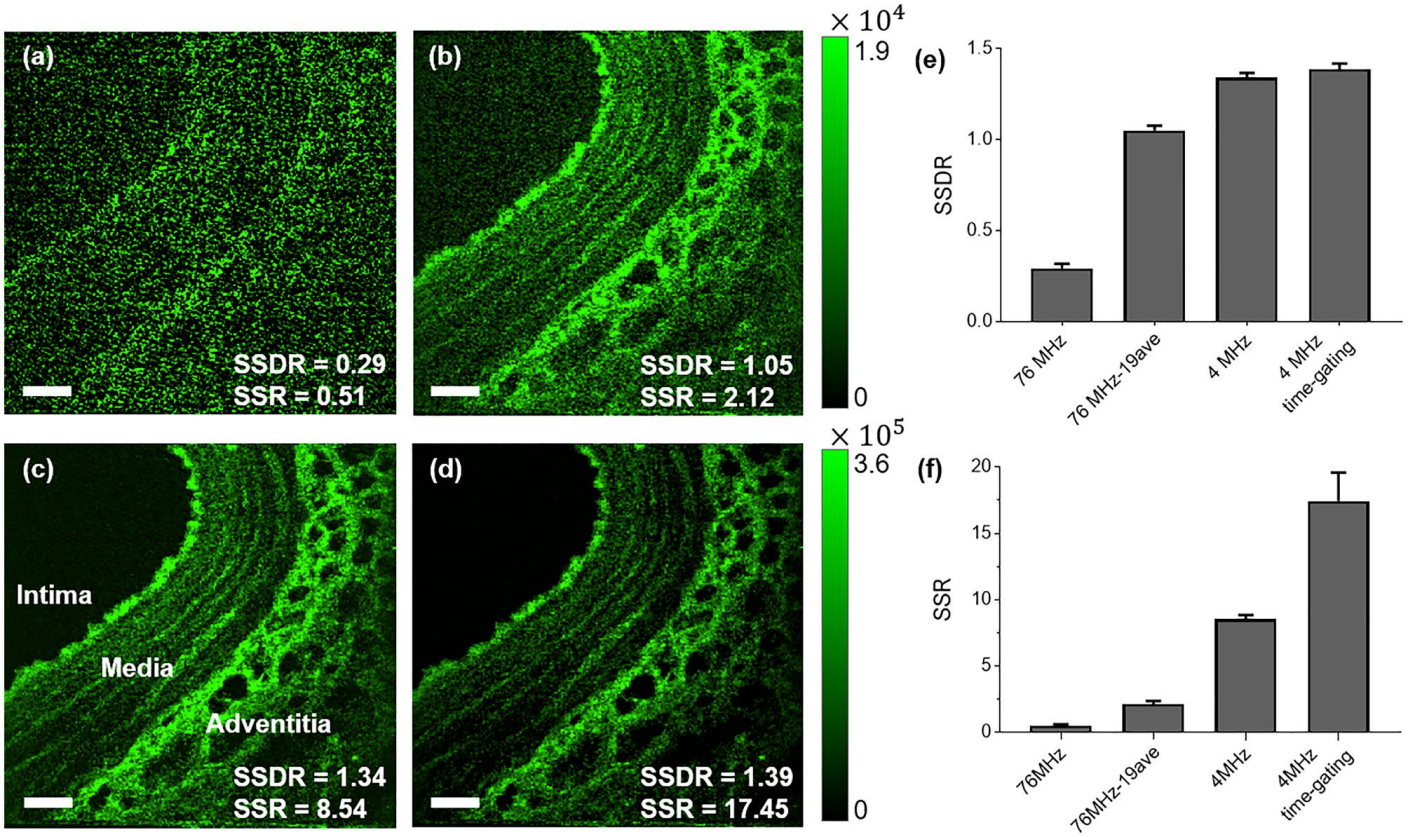
Two-photon fluorescence intensity imaging of the swine coronary artery slice (30 µm thickness) with a pulse repetition rate of (**a**) 76 MHz, (**b**) 76 MHz with 19 frames averaged, (**c**) 4 MHz, and (**d**) 4 MHz and time gating detection method. Note that fluorescence intensity is color-coded in green, and the intensity scale has been adjusted to show a wider range of intensity. The scale bars in all images are 50 µm. The bar charts with standard deviation show (**e**) the signal to both standard deviation ratio (SSDR), and (f) the signal to standard deviation ratio (SSR) for (**a**–**d**).

Similarly, the tissue section of swine coronary artery was imaged without any staining, since relatively strong autofluorescence can be obtained from elastin-rich tissues^26,48^. Figure 3 shows the two-photon fluorescence images of swine coronary artery with (a) high repetition rate of 76 MHz, (b) high repetition rate of 76 MHz and averaging of successive 19 frames, (c) 4 MHz, and (d) 4 MHz and time gating method with a window size of 12.5 ns. The time gating detection method is not applied to images captured at a repetition rate of 76 MHz (Fig. 3a and b). The on-sample power was the same as 8.5 mW for all experiments. As shown in Fig. 3a–d, we observed the coronary artery section composed of the three layers, intima, media, and adventitia. The intima, which is the innermost layer of coronary artery, is composed of endothelial cells, basement membrane, and internal elastic membrane. The high intensity of fluorescence emission from the elastic membrane can be investigated. The thin elastin fibers are observed in the media layer that is composed of elastin including extracellular matrix (ECM) and smooth muscle cells. The adventitia exterior of media is composed of dense elastin and collagen fibers, especially inner adventitia includes a large amount of elastin fibers as shown in Fig. 3^49–51^.

Figure 3e and f show the bar chart of the SSDR and SSR of the Fig. 3a–d. For measurement of SSDR and SSR, ROI containing elastin fluorescence signal was selected. The mean SSDR of the images are 0.29, 1.05, 1.34 and 1.39, respectively, and the mean SSR of the images are 0.51, 2.12, 8.54 and 17.45, respectively. Since the vascular tissue sample emitted lower autofluorescence signal than the fluorescent slide, the SSDR of Fig. 3a is lower than 1.0, showing that the background noise and fluorescence signal are not well distinguished. Averaging 19 images increases the SSDR 3.58 times as shown in Fig. 3b. In fact, the SSDR of Fig. 3c using a low pulse repetition rate with the same average power is 4.16 times higher than Fig. 3a without averaging. Similar to Fig. 2b and c, the SSDR of Fig. 3b is smaller than that of Fig. 3c due to the non-negligible background noise. In this case, the imaging acquisition time and the average power on sample was the same for Fig. 3a and c. With a pulsed source with a low pulse repetition, high-quality fluorescence image can be obtained with high speed. The SSR of the image using time-gating method using a repetition rate of 4 MHz (Fig. 3d) is 2.04 times higher than Fig. 3c and 34 times higher than Fig. 3a.

### Photobleaching effect of repetition rate

**Figure 4:**
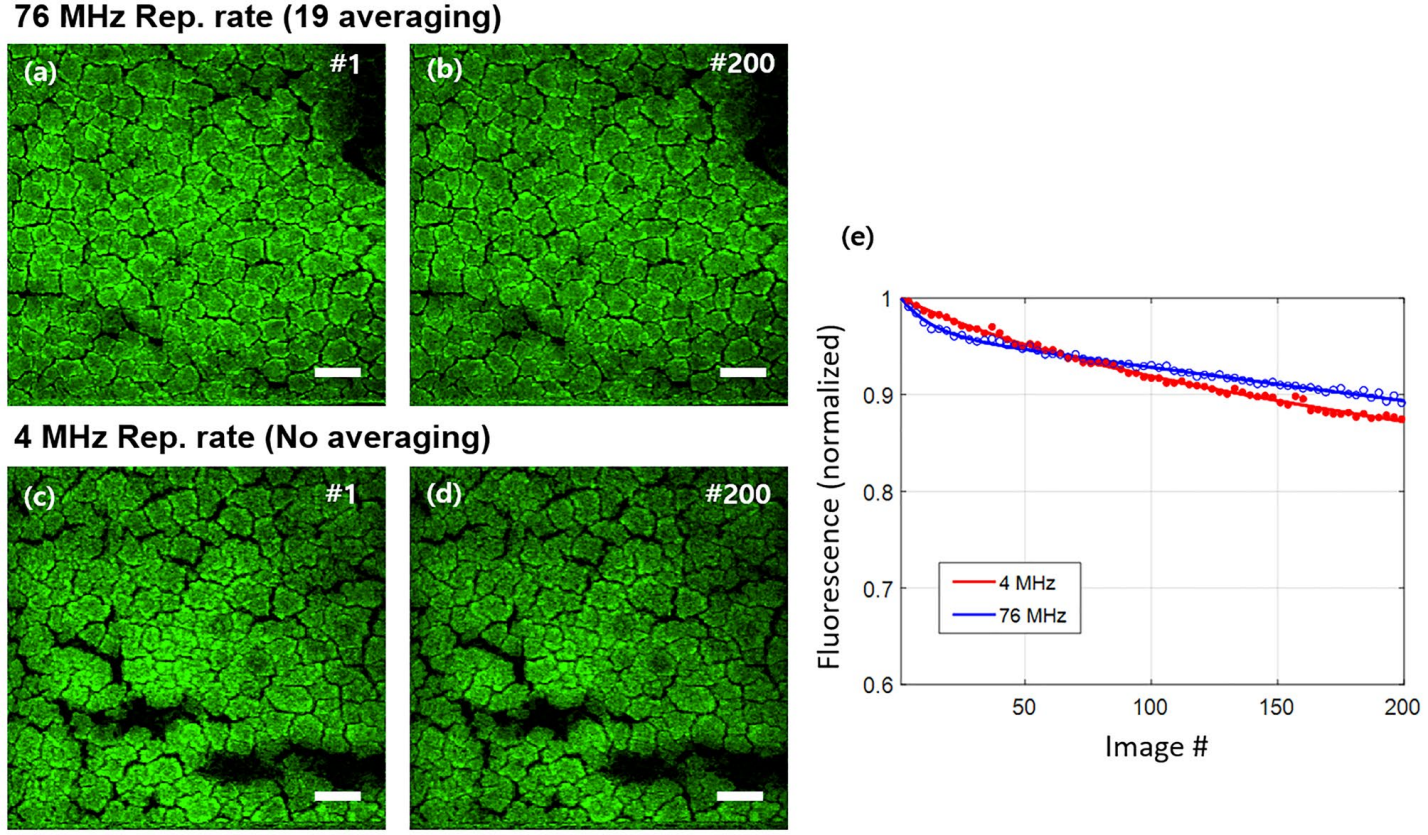
(**a**, **b**) The first and the 200th image of the mouse liver tissue slice (30 µm thickness) irradiated at 76 MHz, respectively. Note that 19 successive frames were averaged to generate a single image to get similar SNR with a single image at 4 MHz. (**c**, **d**) The first and the 200th image of the mouse liver tissue slices irradiated at 4 MHz, respectively, at the same average power. (**e**) The plot of fluorescence intensity versus the acquired image number showing photobleaching. Scale bars 50 µm.

Figure 4a–d show the autofluorescence images of intrinsic fluorophores of mouse liver tissue, such as NADH and FAD^52,53^. While no averaging was needed for 4 MHz pulse repetition rate (Fig. 4c, d), for equivalent SSDR image acquisition, the averaged image was obtained by successively acquiring 19 frames at a 76 MHz pulse repetition rate, which took 19 times longer for a single averaged image (Fig. 4a, b). The single averaged image using a 76 MHz repetition rate was obtained at a 2.5 s, and a single image using 4 MHz repetition rate was obtained at 0.13 s, which was about 19 times faster. The average power was the same as 8.5 mW for both 4 MHz and 76 MHz. The 200 autofluorescence images of mouse liver tissue slices were successively acquired using 76 MHz (Fig. 4a,b) and 4 MHz pulsed source (Fig. 4c,d).

The fluorescence decrease due to the photobleaching effect was measured by summation of image pixel intensities, as shown in Fig. 4e. Fluorescence intensity of the 1st image was normalized to 1 for each photobleaching experiments. The fluorescence decay curve can be fitted to a double exponential equation^54–57^. Fluorescence intensity was decreased by 10.6% and 12.8% during continuous 200 images acquisition at 76 MHz and 4 MHz, respectively, with the same averaging power. Although the photobleaching effect was slightly severe for 4 MHz, significant photobleaching was not observed for both cases. Of note, the imaging time for 76 MHz was 19 times longer due to the averaging to achieve the same SSDR. Therefore, the total energy illuminated for 76 MHz images was 19 times more than 4 MHz images, so there is a lower risk of thermal damage for 4 MHz. In this experiment, when a low repetition rate was used, the nonlinear photobleaching effect caused by high pulse energy remained similar to that of image acquisition using a conventional source with a high repetition rate during the acquisition of 200 images.

## Discussion

TPM often suffers from low SNR. In case of label-free autofluorescence imaging, this matter is more important due to relatively low fluorescence emission compared to exogenous fluorochromes. Of course, averaging multiple frames can improve the SNR, but with a longer light exposure to samples for a long time. In addition, increasing average illumination power can also enhance SNR of two-photon images. However, the drawbacks of these methods include the long acquisition time and high phototoxicity.

In this study, to resolve these problems, we developed a SNR-enhanced high-speed TPM with a pulsed laser having a low pulse repetition rate and time gating detection method. Specifically, simply by adding a pulse picker, we reduced the pulse repetition rate of a Ti:Sapphire laser from 76 to 4 MHz. In spite of using the same average power on the sample, higher two-photon fluorescence emission can be obtained thanks to the quadratic relationship of two-photon absorption. Without increasing potential heat damage, which can be caused by high average power, the pulsed source with low repetition rate allows us to acquire SNR-enhanced two-photon images in a short acquisition time.

The imaging studies using the fluorescent slide and the swine vascular tissue slide at a pulse repetition rate of 76 MHz and 4 MHz were demonstrated. In addition, applying the time gating detection method with a 12.5–25 ns width of window removed background noise that significantly increased SNR of fluorescence images. We demonstrated that we can achieve either higher SNR (Figs. 2c,d vs. 2a and Figs. 3c,d vs. 3a) or higher imaging speed (Figs. 2c,d vs. 2b and Figs. 3c,d vs. 3b) by reducing the pulse repetition rate and applying time gating. Figure 1a,b, which are based on Eqs. (14) and (15), show that the SSDR of 19 averaged images with a 76 MHz repetition rate is expected to be the same as the SSDR of the images with a 4 MHz repetition rate with the same average power. This means that the same SSDR can be obtained if the same amount of fluorescence emission is detected. However, we found that we can achieve both higher SNR and higher speed (Figs. 2c vs. 2b, and Figs. 3c vs. 3b) by reducing repetition rate. In fact, the SSDR of images with a 4 MHz repetition rate (Figs. 2c and 3c) is higher than that of 19 averaged images (Figs. 2b and 3b) with a 76 MHz repetition rate. This result is because the background noise of Eq. (9) was not small enough to be completely ignored in these two-photon fluorescence images. A higher SNR can be achieved by using a low pulse repetition rate because the impact of background noise is greater on images that are averaged with a high repetition rate. Additionally, we could further enhance SNR by the time gating.

One concern about the presented method was high nonlinear photobleaching due to the high pulse peak power. When photobleaching was evaluated with mouse liver tissue by acquiring 200 images, the fluorescence reduction was similar at both 4 MHz and 76 MHz. In addition, the presented high-speed two-photon microscopy using a low repetition rate can achieve the same SNR with much less illumination energy on sample compared to the conventional approach. One limitation of the current study is that it provides only images of static samples, while demonstrating the imaging of dynamic live samples would further highlight the impact of the proposed method. We specifically chose sections of swine coronary artery and mouse liver tissue for a direct quantitative comparison between the conventional method and the proposed method, given the inherent challenges in quantitatively comparing dynamic live samples. Nonetheless, the successful demonstration of autofluorescence imaging in the biological samples indicates the potential adoption of the proposed method in live sample imaging studies without significant issues.

In conclusion, instead of conventional repetition rate of 76 or 80 MHz of Ti:Sapphire laser, by decreasing the repetition rate of the pulse laser of the TPM, higher SNR images can be acquired at a faster rate, without too much concern of increasing the photobleaching problem. We anticipate that the developed TPM with a pulsed laser with low repetition rate will offer significant advantages in achieving fast, high-SNR imaging.

## Method

### Characterization of the two-photon microscope system with a controllable repetition rate

**Figure 5:**
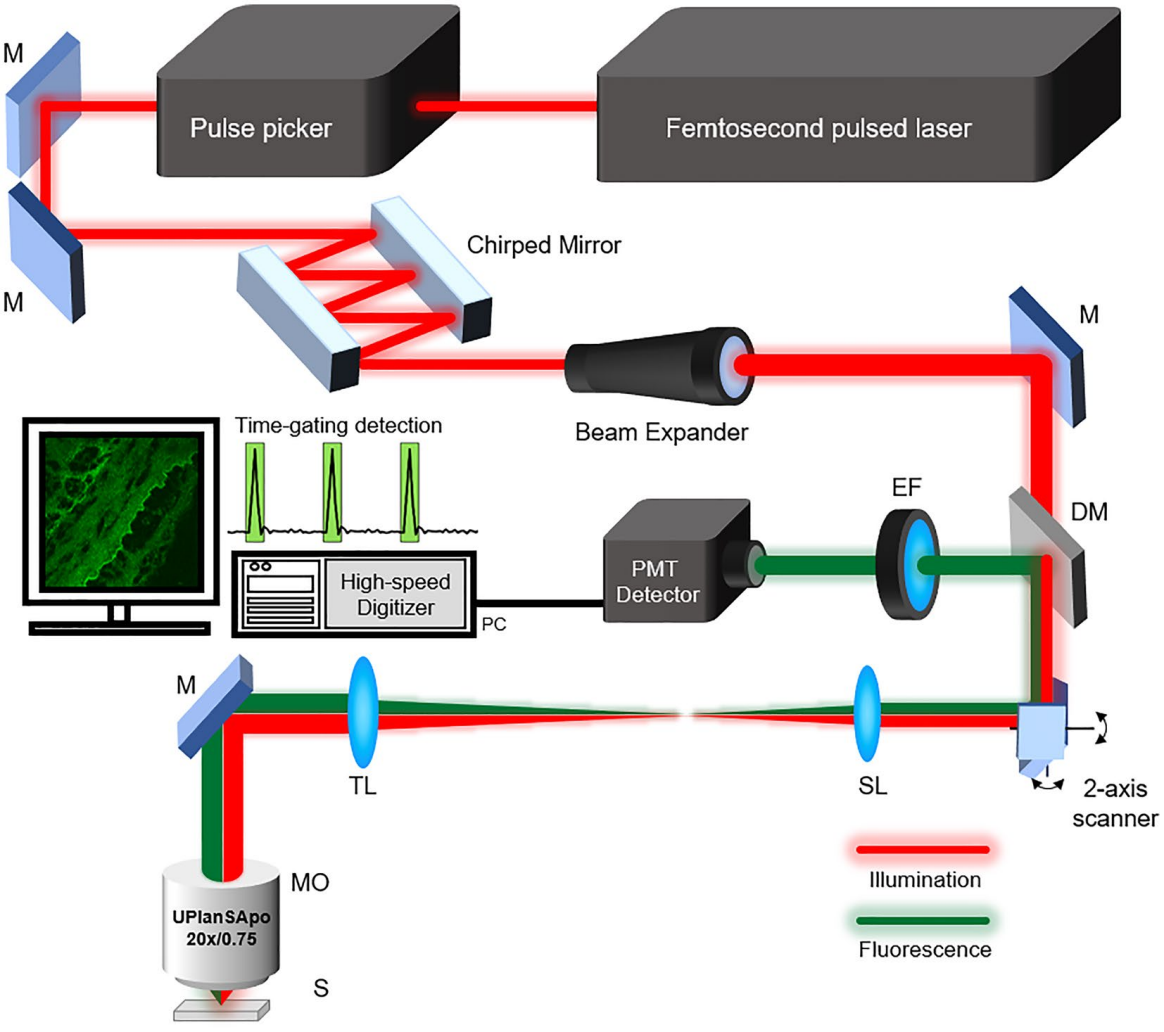
Schematic diagram of a two-photon microscope (TPM) with a controllable repetition rate. A pulse picker installed after the femtosecond pulsed laser can control the pulse repetition rate. (M = mirror, DM = dichroic mirror, SL = scan lens, TL = tube lens, MO = microscope objective, EF = emission filter, S = sample).

Figure 5 shows the schematic diagram of the two-photon microscope system used in this study. A wavelength tunable femtosecond pulsed laser (Mira 900-f, Coherent, USA) was tuned to have a center wavelength of 816 nm. The repetition rate of the pulsed laser source itself was 76 MHz. We used a pulse picker (Pulse Select, APE, Germany) to control the repetition rate of the femtosecond pulsed laser. As the pulse picker selectively picks out pulses from the 76 MHz repetition rate source, we can select repetition rates corresponding to the number obtained by dividing 76 MHz by an integer. A pair of chirped mirrors (DCMP 175, Thorlabs, USA) were used to pre-compensate the group delay dispersion (GDD) induced by the optics in the beam path so the pulse would remain narrow at the sample for high peak power. The pulse widths for different repetition rates were measured using an autocorrelator (Mini, APE, Germany) and were maintained to be 139 femtoseconds. A beam expander (GBE03-B, Thorlabs, USA) was used to expand the beam size to fully fill the back aperture of the objective lens. A galvano mirror (6220H, Cambridge Technology, USA) and 4 kHz resonant scanner (CRS 4 kHz, Cambridge Technology, USA) were used to scan the beam. A scan lens with a focal length of 50 mm (f100-100 pair of #49-360-INK, Edmund Optics, USA) and a tube lens with a focal length of 200 mm (ITL200, Thorlabs, USA) were used as relay optics to adequately guide the scanned beam into the objective lens (UPlanSAPO 20x/0.75, Olympus, Japan).

Under the pulsed excitation by the laser source, two-photon fluorescence emission signal from the sample was reflected by a dichroic mirror (ff705-di01, Semrock, Rochester, NY). An emission filter (FF01-550/200, Semrock, Roshcester, NY) was used to select the fluorescence emission. A fiber collimator (PAF2P-11A/15B, Thorlabs, NJ) was used to couple the fluorescence signal into the multimode fiber (FVP400440480, Molex, Lisle, IL), which was directed to the high-sensitive photomultiplier detector (PMT, H10721-20, Hamamatsu, Photonics, Japan). The detected signal was amplified by a current-to-voltage amplifier with a bandwidth of 50 MHz (C6438-01, Hamamatsu, Photonics, Japan). Then a digitizer with a sampling rate of 400MS/s (PX14400D, Signatec, IL) digitized and recorded the amplified fluorescence signal. For synchronization, the pulse signal of the pulse picker and the trigger output of the resonant scanner are combined and utilized as a sync signal of the galvano and digitizer. Data series of signals are transferred to the computer and processed by our imaging software programmed in C++. The software displays two-photon images in real-time.

### Data acquisition and processing

Our TPM acquired the fluorescence images with FOV of 400 × 400 ${\mu m}^{2}$ composed of 512 × 512 pixels. In the experiment, 4 MHz and 76 MHz repetition rate pulsed sources were used for comparison. Using a pulsed excitation with a repetition rate of 4 MHz, a single excitation pulse creates a single pixel by applying time gating on 100 data samples acquired by the high-speed digitizer during 250 ns of pixel dwell time. In the pulsed excitation with a repetition rate of 76 MHz, 19 excitation pulses were used to create a single pixel by accumulating 100 data samples acquired by the high-speed digitizer during 250 ns. The average power was maintained by using a pulse picker electronic interface. For comparing SSDR and SSR, same positions of samples were analyzed. The SSDR and SSR were measured by using a raw data of image intensity with MATLAB.

For photobleaching effect measurement, label-free mouse liver tissue slices were irradiated with a pulse repetition rate of 76 MHz and 4 MHz at the same average power. When 76 MHz repetition rate was used, 19 successive images were averaged to create an image, which has the similar SSDR to the image with 4 MHz repetition rate. 200 consecutive images were obtained for both cases to analyze the photobleaching effect, respectively. By measuring the total amount of fluorescence in the images, the fluorescence reduction ratio caused by photobleaching was calculated.

### Time gating method for background noise reduction

**Figure 6:**
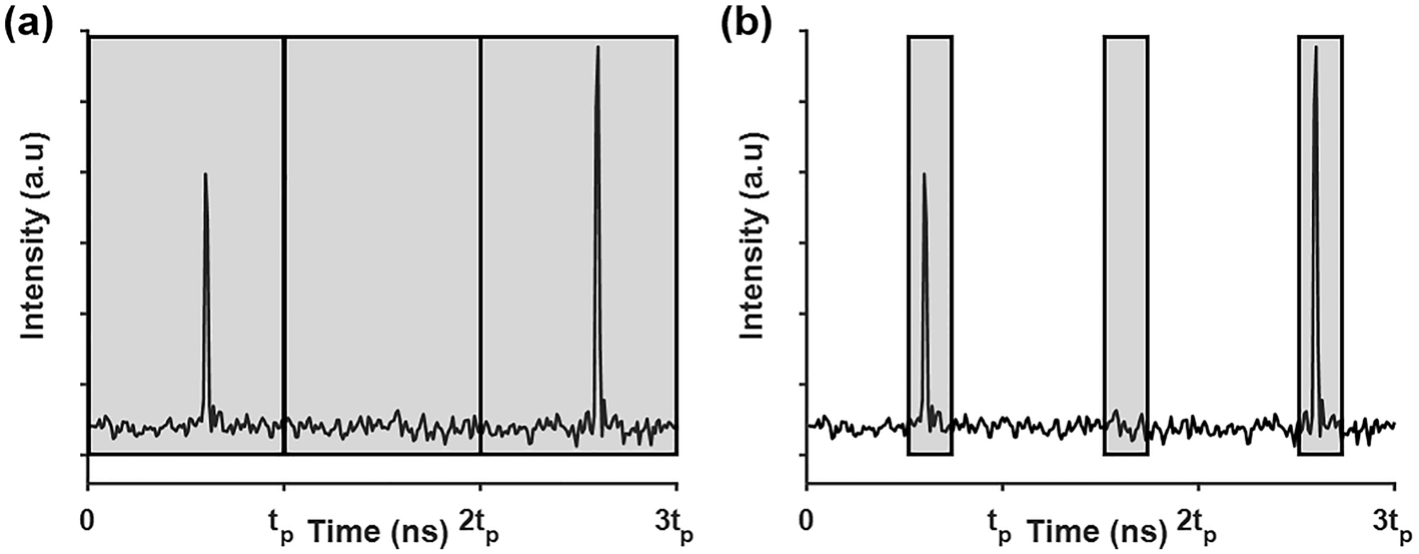
Schematics of (**a**) a method of determining pixel value by integration of digitized data during pixel dwell time and (**b**) the proposed time gating detection method.

Figure 6a shows a commonly used method to determine a pixel intensity using sampling data during pixel dwell time. All sampling data series in pixel dwell time are used to calculate pixel intensity. In this study, we present the time gating detection method in the two-photon imaging to eliminate background noise detection as shown in Fig. 6b. The size of the time window was determined by measuring the full width of the pulsed fluorescence signal that can contain the entire fluorescence signal without background noise. Integration of signals within the time gates adjusted for the fluorescence intensity can remove a significant amount of background noise. With a low repetition rate pulsed source, we can apply the smaller number of time gates in signal detection to reduce background noise, resulting in higher SSR and SSDR of two-photon images.

### Imaging sample preparation

A fluorescent slide sample (FSK2, Thorlabs, USA) was used as an imaging sample to validate the SNR increase of two photon images. The fluorescent slide provided uniform two-photon signals across all imaging field. The biological tissue samples, swine coronary artery and mouse liver tissue, were used as imaging samples to validate SNR enhancement and to estimate photobleaching effect. Swine coronary artery section was obtained from a Yucatan miniature swine (male, 3 months old, 15–20 kg; Optipharm Co., Ltd., Korea). Mouse liver tissue section was obtained from a BALB/c mouse at 6 weeks of age were fed the normal diet for 10 weeks (Central Lab. Animal Inc., Korea). Swine was euthanized under anesthesia using potassium chloride (KCL, 2 mmol/kg). Mouse was euthanized under anesthesia by ${\mathrm{CO}}_{2}$ inhalation. Using a cryotome, the excised biological tissues were sectioned to 30 µm and attached to a slide glass without any labeling. This study was approved by the Institutional Animal Care and Use Committee (IACUC) of the Korea University College of Medicine (KOREA-2018-0070 and KOREA-2021-0076). All the methods are confirmed in accordance with the ARRIVE guidelines and regular guidelines and regulations.

## Data Availability

The datasets generated and/or analysed during the current study are available from the corresponding author on reasonable request.
